# EV-D68 cleaves LARP1 and PABPC1 by 3C^pro^ to redirect host mRNA translation machinery toward its genomic RNA

**DOI:** 10.1371/journal.ppat.1013098

**Published:** 2025-04-28

**Authors:** Ruyang Tan, Yuling Zhang, Mengqian Huang, Honghua Chen, Zixiang Liu, Zining Wang, Xiaoyan Li, Tao Wang, Zhiyun Wang

**Affiliations:** 1 School of Life Sciences, Tianjin University, Tianjin, China; 2 Tianjin Centers for Disease Control and Prevention, Tianjin, China; 3 Tianjin Key Laboratory of Pathogenic Microbiology of Infectious Disease, Tianjin, China; 4 School of Environmental Science and Engineering, Tianjin University, Tianjin, China; Stanford University, UNITED STATES OF AMERICA

## Abstract

Enterovirus D68 (EV-D68) is an emerging pathogen associated with severe respiratory diseases and neurological complications, such as acute flaccid myelitis. EV-D68 has developed sophisticated mechanisms to hijack host translation machinery, facilitating its replication and impairing host mRNA translation. In this study, we demonstrate that EV-D68 cleaves La-related protein 1 (LARP1) and poly(A)-binding protein cytoplasmic 1 (PABPC1) through its proteases 3C^pro^ and 2A^pro^. Our results indicate that overexpressing LARP1 and PABPC1 significantly inhibits EV-D68 replication and reduces the virus-mediated suppression of host translation. While both LARP1 and PABPC1 regulate translation, they exert antiviral effects through distinct mechanisms. We found that LARP1 interacts with the 5’UTR of EV-D68 RNA through its LAM domain, and this interaction is crucial for its antiviral function. LARP1 translation modulation is also influenced by the mTOR and CDK1 signaling pathways. Viral infection inhibits mTOR and CDK1 phosphorylation, which enhances LARP1’s binding to viral RNA and inhibits viral translation. To counteract this inhibition, EV-D68 cleaves LARP1 through 3C^pro^, thereby promoting efficient viral translation. We also investigated other enteroviruses, such as EV-A71 and CV-A16, which similarly target LARP1 and PABPC1, indicating a conserved mechanism across enteroviruses. Our findings offer new insights into how EV-D68 manipulates host translation and highlight the potential of targeting LARP1 and PABPC1 for antiviral interventions.

## Summary

Enterovirus D68 (EV-D68) is a respiratory virus that has been linked to severe respiratory illnesses and neurological conditions, such as acute flaccid myelitis. Like many viruses, EV-D68 hijacks the host’s protein-making machinery to promote its own replication while suppressing the production of host proteins. In our study, we explored how EV-D68 interacts with a key host protein, La-related protein 1 (LARP1), which plays an important role in controlling protein production in human cells. We found that EV-D68 specifically targets and cleaves LARP1 using one of its proteases (3C^pro^). This cleavage disrupts LARP1’s ability to bind to viral genetic material, allowing the virus to bypass the host’s natural defense mechanisms and efficiently produce its own proteins. Additionally, we discovered that this process is linked to major cellular signaling pathways, which regulate how proteins are made in the cell. By interfering with these pathways, EV-D68 enhances its ability to replicate and spread. Our findings shed light on how EV-D68 manipulates host cells for its survival and identify LARP1 as a potential target for antiviral strategies. Understanding these interactions could help in the development of treatments that limit EV-D68 infection and its associated diseases.

## Introduction

Enterovirus D68 (EV-D68), a member of the *Picornaviridae* family, is a non-enveloped, single-stranded RNA virus with a virion diameter of approximately 30 nm [1]. First isolated in 1962 from children with pneumonia and bronchiolitis in California, EV-D68 has since become a significant pathogen globally, especially affecting children. Unlike most enteroviruses, EV-D68 primarily causes respiratory illnesses, including pneumonia, bronchiolitis, and asthma, with symptoms similar to the common cold, such as runny nose, cough, fever, and muscle aches [2]. Notably, EV-D68 spreads through respiratory droplets rather than the fecal-oral route, distinguishing it from many other enteroviruses [3–6]. Since its resurgence in 2005, EV-D68 outbreaks have become more frequent, with a notable increase in cases during the 2014 U.S. outbreak and a rise in infections during the SARS-CoV-2 pandemic [2,7–9]. This has raised concerns over the virus’s potential threat to global public health, particularly due to its association with severe respiratory symptoms and central nervous system complications, such as acute flaccid myelitis (AFM) [10]. Despite efforts to identify antiviral drugs and develop vaccines, no effective therapies, or vaccines for EV-D68 have advanced beyond preclinical stages [11–15].

The La-Related Protein 1 (LARP1) is an evolutionarily conserved RNA-binding protein (RBP) of the LARP family that plays a vital role in translation regulation. LARP1 exists in two common isoforms, which share high sequence conservation in the LAM, RRM-L5, and DM15 domains but differ in their promoter usage [16]. LARP1 interacts with the 3′ poly(A) tail and the 5′-terminal oligopyrimidine tract (5′TOP) of mRNAs, regulating mRNA translation in response to cellular nutrient conditions [17]. Under nutrient-rich conditions, mTOR signaling activates the phosphorylation of LARP1, causing it to dissociate from mRNA 5′ untranslated regions (5′UTRs) and enhance translation. In contrast, nutrient deprivation inhibits mTOR phosphorylation, preventing LARP1 dissociation, thereby reducing translation initiation [18–20]. LARP1 phosphorylation is also regulated by cyclin-dependent kinase 1 (CDK1), which links cellular proliferation to mRNA translation [21]. Although LARP1’s role in translation regulation is well-documented, its involvement in viral infections is not fully understood. Studies have shown that LARP1 interacts with viral RNA and affects viral replication in infections such as hepatitis C virus (HCV), dengue virus (DENV), and SARS-CoV-2, but its role in EV-D68 infection remains unexplored [22–24].

EV-D68 utilizes a type I internal ribosome entry site (IRES) in its 5′UTR, allowing cap-independent translation of viral RNA [25–28]. During infection, enteroviruses, including EV-D68, hijack host translation by cleaving key translation initiation factors using viral proteases. EV-D68’s 2A protease (2A^pro^) cleaves eIF4G, a crucial component of the eIF4F complex that facilitates translation initiation [29]. The cleavage of eIF4G prevents cap-dependent translation, but the remaining C-terminal fragment can still support IRES-driven translation of viral RNA, allowing viral mRNA translation while suppressing host translation [30]. Several antiviral strategies have been explored to target viral proteases and IRES structures. For example, studies have shown that Human enterovirus 71 (EV-A71) 2A^pro^ and 3C protease (3C^pro^) enhance IRES-dependent translation, prompting research into protease-targeting antiviral strategies [31]. Notably, small molecules such as 3-benzyl coumarin derivatives have been reported to counteract the inhibitory effects of EV-A71 2A^pro^ on host translation, ultimately reducing viral replication [32]. Additionally, Rupintrivir, a 3C^pro^ inhibitor that has progressed to clinical trials, has shown efficacy against multiple viruses, including rhinoviruses, noroviruses, EV-A71, Coxsackievirus A16 (CV-A16), SARS-CoV-1, and SARS-CoV-2, underscoring the potential of protease inhibitors as a therapeutic strategy against enteroviral infections [33–39].

While enteroviruses have well-established mechanisms to hijack host translation by targeting core initiation factors, it remains unclear whether EV-D68 can also affect other translation-related proteins to compete for translation machinery. In this study, we demonstrate that EV-D68 uses its proteases, 3C^pro^ and 2A^pro^, to cleave translation-related proteins LARP1 and PABPC1, thereby suppressing host mRNA translation and commandeering host translation machinery to promote viral replication. Our findings elucidate a novel mechanism by which EV-D68 manipulates host translation and provide new insights into the broader impact of enteroviruses on host translation, highlighting potential avenues for antiviral therapy development.

## Results

### Viral cleavage of LARP1 and PABPC1 inhibits host eukaryotic translation

**Fig 1:**
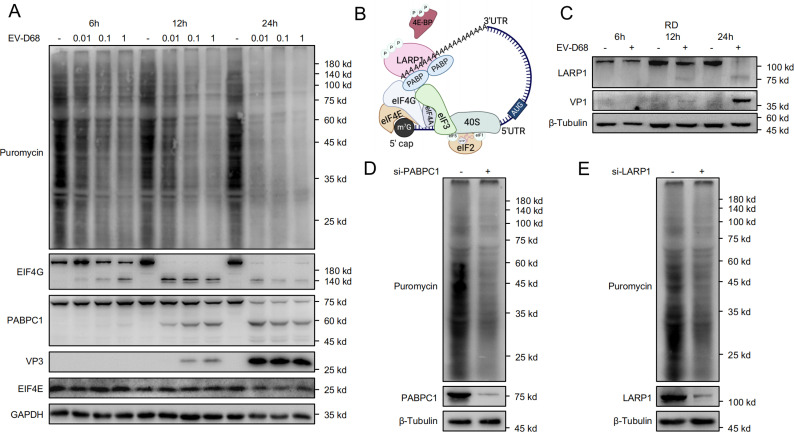
Viral Cleavage of LARP1 and PABPC1 Inhibits Host Eukaryotic Translation. (A) RD cells were infected with EV-D68 at MOIs of 0.01, 0.1, and 1, and harvested at 6 h, 12 h, and 24 h. Cells were labelled with 10 µg/mL puromycin and treated with puromycin for 0.5 h. Cells were collected for western blot. (B) A model of the translation initiation complex, including eIF4E, eIF4G, LARP1, and PABPC1. Created in BioRender. Ruyang, T. (2025) https://BioRender.com/q79y826. (C) RD cells were infected with EV-D68 (MOI = 0.1) for 6 h, 12 h, and 24 h, and then collected for western blot. (D) RD cells were transfected with negative control (NC) or pooled PABPC1 siRNA for 48 h. Cells were labelled with 10 µg/mL puromycin for 0.5 h, and then collected for western blot. (E) RD cells were transfected with NC or pooled LARP1 siRNA for 48 h. Cells were labelled with 10 µg/mL puromycin for 0.5 h and then collected for western blot.

It is well established that enteroviruses can hijack host cellular machinery to facilitate their own proliferation while inhibiting host mRNA translation. To investigate whether EV-D68 shares this ability to suppress host translation, RD cells were infected with EV-D68 at MOI = 0.01, 0.1, and 1 for 6, 12, and 24 hours, followed by puromycin labeling to quantify global mRNA translation (Fig 1A). Puromycin is a structural analogue of the aminoacyl-tRNA, which is incorporated into nascent polypeptide chains and can be detected via immunoblotting, providing a measure of mRNA translation in cells [40,41]. Our result shows a dose- and time-dependent reduction in puromycin incorporation, indicating a significant inhibition of host translation by EV-D68.

To understand how this inhibition might occur, we constructed a model of the translation initiation complex, highlighting key host proteins involved in translation initiation, including eIF4E, eIF4G, LARP1, and PABPC1 (Fig 1B). Enteroviruses have previously been shown to cleave eIF4G to disrupt cap-dependent translation [42–44]. In our study, we observed that eIF4G cleavage occurred as early as 6 hours post-infection, coinciding with a decrease in puromycin incorporation. However, at 12 hours post-infection, even though eIF4G cleavage was nearly complete, global mRNA translation continued to decline (Fig 1A).

Previous studies have reported that PABPC1 is cleaved by other enteroviruses, such as Poliovirus and Coxsackievirus to inhibit host mRNA circularization and cap-dependent translation and facilitate viral replication [45,46]. To test whether EV-D68 similarly affects PABPC1, we analyzed PABPC1 protein levels in infected cells, as shown in Fig 1A. Western blot analysis revealed a dose- and time-dependent cleavage of PABPC1 in EV-D68-infected cells, with clear cleavage bands observed, consistent with the pattern observed in other enteroviruses [46–48].

We further analyzed the impact of EV-D68 on eIF4E, another key component of the translation initiation complex. Unlike eIF4G and PABPC1, eIF4E protein levels remained unchanged upon EV-D68 infection (Fig 1A). These observations indicate that PABPC1 cleavage is associated with suppression of host protein synthesis and enhanced viral replication.

Numerous studies have shown that PABPC1 and LARP1 interact directly, forming a complex with RNA to facilitate mRNA stabilization and translation [49,50]. To validate this interaction, we performed co-immunoprecipitation and Duolink proximity ligation assays (PLA). Our results confirmed the interaction between LARP1 and PABPC1 (S1A, B Fig), supporting the studies that these proteins act in concert to regulate translation. Given their interaction, we hypothesized that EV-D68 might also target LARP1 to further impair host translation.

Building upon these findings, we infected RD cells with EV-D68 at MOI = 0.1 and collected samples at 6, 12, and 24 hours post-infection for Western blot and qPCR analysis (Fig 1C). Consistent with our hypothesis, LARP1 protein levels decreased significantly over time, with evidence of cleavage bands appearing at 75 kDa. Interestingly, while LARP1 protein levels decreased, LARP1 mRNA levels increased in response to infection (S1C Fig), suggesting that the host may upregulate LARP1 transcription in response to protein cleavage. Furthermore, we observed a similar cleavage pattern of LARP1 in HEK293T cells, indicating that EV-D68-mediated LARP1 cleavage is not specific to RD cells (S1D Fig).

Finally, to simulate the viral cleavage of PABPC1 and LARP1, we used siRNA to knock down each protein individually in uninfected cells, aiming to determine whether their downregulation affects host translation. Puromycin labeling revealed a marked decrease in mRNA translation upon PABPC1 or LARP1 knockdown (Fig 1D, E), mirroring the effects of viral infection. These results suggest that both LARP1 and PABPC1 are crucial for maintaining host translation, and their cleavage by EV-D68 may impair host translation.

### EV-D68 cleaves PABPC1 and LARP1, inhibiting eIF4E-cap-dependent mRNA translation

Previous studies have shown that enteroviral proteases 2A^pro^ and 3C^pro^ target and cleave PABPC1, disrupting its interaction with the poly(A) tail and thereby impairing host mRNA translation, which ultimately inhibits host translation [47]. In this study, we examined whether EV-D68 also utilizes a similar mechanism to modulate host translation.

**Fig 2:**
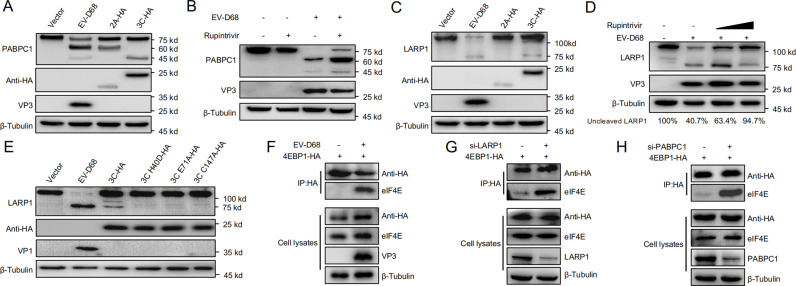
EV-D68 Cleaves PABPC1 and LARP1, Inhibiting eIF4E-Cap-Dependent mRNA Translation. (A) Empty Vector, 2A-HA, and 3C-HA plasmids were transfected into RD cells for 24 h, and then EV-D68 was infected for 24 h. Cells were then collected for western blot. (B) RD cells were infected with EV-D68, and Rupintrivir (10 µ M) was added at 6 h post-infection for a total of 18 h. Cells were harvested at 24 h post-infection for western blot analysis. (C) Empty Vector, 2A-HA, and 3C-HA plasmids were transfected into RD cells for 24 h, and then EV-D68 was infected for 24 h. Cells were then collected for western blot. (D) RD cells were infected with EV-D68, and treated with Rupintrivir (5 µ M or 10 µ M) starting at 6 h post-infection for 18 h. Cells were collected at 24 h post-infection for western blot analysis. (E) Empty Vector, 3C-HA, 3C-H40D-HA, 3C-E71A-HA, 3C-C147A-HA plasmids were transfected into RD cells for 24 h, then EV-D68 infected control cells for 12 h. Cells were harvested for western blot. (F) HEK293T cells were transfected with 4EBP1-HA plasmid for 24 h, infected with EV-D68 (MOI = 0.1) for 12 h, and cells were collected for Co-IP. (G) HEK293T cells were transfected with NC or PABPC1 siRNA for 24 h, transfected with 4EBP1-HA plasmid for 24 h, and cells were collected for Co-IP. (H) HEK293T cells were transfected with NC or LARP1 siRNA for 24 h, transfected with 4EBP1-HA plasmid for 24 h, and cells were harvested for Co-IP.

Empty vector was used as a control, while 2A-HA and 3C-HA plasmids encoding EV-D68 proteases were transfected into RD cells using EV-D68-infected cells as a positive control [51] (Fig 2A). Immunoblotting showed that EV-D68 infection resulted in prominent PABPC1 cleavage bands at 60 kDa and 45 kDa, while transfection with 2A^pro^ resulted in a cleavage band at 60 kDa and transfection with 3C^pro^ resulted in a band at 45 kDa, suggesting that both 2A^pro^ and 3C^pro^ are involved in PABPC1 cleavage. Treatment with the 3C^pro^-specific inhibitor Rupintrivir partially restored PABPC1 levels [52,53], indicating that 3C^pro^ activity was inhibited but suggesting that 2A^pro^ also plays a role in PABPC1 cleavage (Fig 2B).

To determine which EV-D68 proteins are involved in LARP1 cleavage, we performed similar experiments to those mentioned above involving RD cells. We observed a prominent LARP1 cleavage band (75 kDa) only in cells transfected with 3C^pro^ or infected with EV-D68, while no such band was observed in 2A-transfected cells. This indicates that 3C^pro^, but not 2A^pro^, is involved in LARP1 cleavage (Fig 2C). We then treated RD cells infected with EV-D68 with increasing concentrations of Rupintrivir and observed a dose-dependent restoration of full-length LARP1 (120 kDa) levels. Densitometric analysis showed that at the highest concentration, LARP1 cleavage was significantly reduced compared to untreated cells, with full-length LARP1 levels restored to 94.7%, indicating effective inhibition of 3C^pro^ activity (Fig 2D). To further confirm the role of 3C^pro^, we mutated three active cleavage sites of 3C^pro^ (H40D, E71A, and C147A) to construct point mutant plasmids, which were co-transfected with Empty Vector and 3C-HA into RD cells, with EV-D68-infected cells serving as a positive control. Immunoblotting analysis showed that LARP1 cleavage bands were observed in cells transfected with wild-type 3C^pro^ and in EV-D68-infected cells, while no cleavage bands were detected in cells transfected with the mutant 3C^pro^ constructs, indicating that all three active cleavage sites of 3C^pro^ are required for LARP1 cleavage (Fig 2E).

To determine the reason why EV-D68 cleaves PABPC1 and LARP1 to inhibit host translation, we performed additional experiments. We transfected HEK293T cells with the cap-binding protein inhibitor 4EBP1-HA, followed by EV-D68 infection (MOI = 0.1) after transfection, and collected the cells for Co-Immunoprecipitation (Co-IP). The results showed that the interaction between the translation inhibitor 4EBP1 and cap-binding protein eIF4E was enhanced in EV-D68-infected cells, indicating that eIF4E’s ability to bind capped mRNA was inhibited, thereby suppressing cap-dependent translation (Fig 2F).

Subsequently, PABPC1 or LARP1 was knocked down in HEK293T cells, followed by transfection of 4EBP1-HA for Co-IP. The results showed that knocking down PABPC1 or LARP1 enhanced the interaction between 4EBP1 and eIF4E, resulting in inhibition of cap-dependent translation (Fig 2G, H). These results indicate that EV-D68 inhibits cap-dependent translation by cleaving PABPC1 or LARP1, thereby reducing eIF4E’s binding to capped mRNA and ultimately suppressing host translation.

### PABPC1 and LARP1 disrupt EV-D68 replication

**Fig 3:**
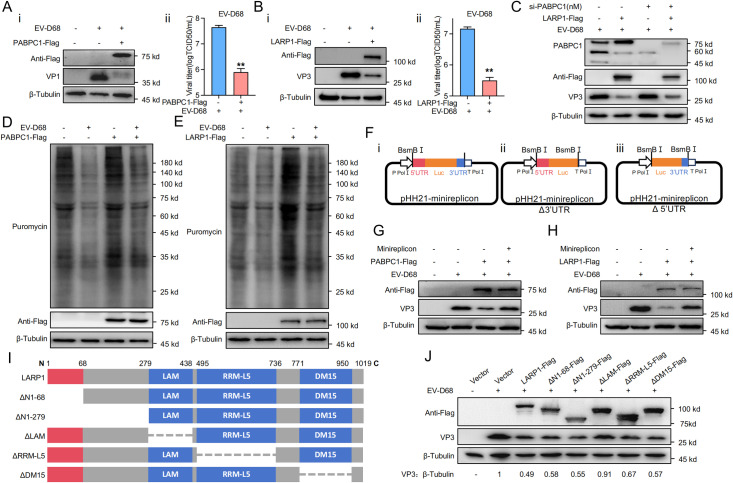
PABPC1 and LARP1 Disrupt EV-D68 Replication. (A) PABPC1-Flag plasmid was transfected in RD cells for 24 h, then infected with EV-D68, and the lysates of the cells were collected after 24 h for western blot (i), and viral titer analysis (ii). (B) LARP1-Flag plasmid was transfected in RD cells for 24 h, then infected with EV-D68, and the lysates of the cells were collected after 24 h for western blot (i), and viral titer analysis (ii). (C) NC or pooled PABPC1 siRNA was transfected in RD cells for 24 h, then transfected with LARP1-Flag plasmid for 24 h, followed by infection with EV-D68 virus for 24 h. Finally, cells were collected for western blot. (D) PABPC1-Flag plasmid was transfected in RD cells for 24 h, then infected with EV-D68, and 12 h later the cells were labelled with 10 µg/mL puromycin for 0.5 h. Cells were then collected for western blot. (E) LARP1-Flag plasmid was transfected in RD cells for 24 h, then infected with EV-D68, and 12 h later the cells were labelled with 10 µg/mL puromycin for 0.5 h. Cells were then collected for western blot. (F) Schematic representation of EV-D68 minireplicon (i); Schematic representation of EV-D68 minireplicon Δ3′UTR (ii); Schematic representation of EV-D68 minireplicon Δ5′UTR (iii). (G) Empty Vector, PABPC1-Flag and EV-D68 minireplicon were transfected for 24 h, then infected with EV-D68, and cells were collected after 12 h for western blot. (H) Empty Vector, LARP1-Flag and EV-D68 minireplicon were transfected in RD cells for 24 h, then infected with EV-D68, and the cells were collected for western blot after 12 h. (I) LARP1 functional domain diagram. (J) Empty Vector, LARP1-Flag, △1-68-Flag, △1-279-Flag, △LAM-Flag, △RRM-L5-Flag, △DM15-Flag plasmid was transfected in RD cells for 24 h, then infected with EV-D68, and the lysates of the cells were collected after 24 h for western blot.

Previous research has shown that PABPC1 and LARP1 are crucial translation-related proteins cleaved by EV-D68, thereby inhibiting host cap-dependent translation while leaving viral translation unaffected. To further explore the impact of PABPC1 and LARP1 on EV-D68 replication, we overexpressed these proteins in RD cells. RD cells were transfected with PABPC1-Flag or LARP1-Flag plasmids, followed by infection with EV-D68, and the cells were collected for immunoblotting and viral titer analysis. The results showed that PABPC1-Flag or LARP1-Flag overexpression significantly suppressed EV-D68 protein levels and viral titers (Fig 3A, B). Furthermore, gradient transfection of LARP1-Flag showed that increased LARP1 expression reduced the cytopathic effect (CPE) caused by infection, as observed under bright-field microscopy (S2A Fig). These results indicate that both PABPC1 and LARP1 can suppress EV-D68 replication.

To determine whether PABPC1 and LARP1 suppress EV-D68 through their interaction, we transfected RD cells with PABPC1 siRNA, followed by LARP1-Flag plasmid transfection, and then infected the cells with EV-D68. Immunoblotting and qPCR analysis showed that LARP1 could inhibit EV-D68 protein levels regardless of PABPC1 knockdown (Figs 3C; S2B). This suggests that LARP1 and PABPC1 suppress EV-D68 independently through their own biological functions rather than through mutual interaction.

Given that LARP1 and PABPC1 are both translation-related proteins, we hypothesized that overexpression of LARP1 and PABPC1 might enhance host mRNA translation, which would reduce EV-D68’s ability to hijack translation machinery and decrease viral mRNA translation, ultimately affecting viral replication. To verify this hypothesis, RD cells were transfected with PABPC1-Flag plasmid, followed by EV-D68 infection and puromycin treatment, and then collected for immunoblotting analysis. The results showed that overexpression of PABPC1 enhanced host mRNA translation (Fig 3D, lanes 1 and 3), but this enhancement was suppressed upon EV-D68 infection (Fig 3D, lanes 3 and 4), although it remained higher than in the control group without PABPC1 transfection (Fig 3D, lanes 2 and 4). This suggests that overexpression of PABPC1 can weaken EV-D68-mediated inhibition of translation. Similarly, overexpression of LARP1 also had the same effect as PABPC1 (Fig 3E).

The EV-D68 minireplicon system is a key tool for studying interactions between viral RNA and host factors. It was constructed by replacing the coding region of the EV-D68 genome with a firefly luciferase reporter gene while retaining the 5’ and 3’UTRs [54]. These minireplicons, including the full-length EV-D68 minireplicon, a minireplicon lacking the 3’UTR (Δ3’UTR), and a minireplicon lacking the 5’UTR (Δ5’UTR), were previously constructed and preserved by our laboratory (Fig 3F). To further confirm this hypothesis, RD cells were transfected with Empty Vector, PABPC1-Flag, and EV-D68 minireplicon, followed by EV-D68 infection. The results demonstrated that transfection of the EV-D68 minireplicon antagonized the inhibitory effect of PABPC1 on EV-D68 at both protein and RNA levels (Figs 3G; S2C). Similarly, in RD cells transfected with Empty Vector, LARP1-Flag, and EV-D68 minireplicon, the EV-D68 minireplicon antagonized LARP1’s inhibition of EV-D68 replication at both the protein and RNA levels (Figs 3H; S2D). These findings indicate that the EV-D68 minireplicon may hijack eIF4G and other translation initiation factors, leading to suppression of the enhanced eukaryotic mRNA translation capacity mediated by PABPC1 or LARP1, thus antagonizing their antiviral effects.

PABPC1, as a component of the translation initiation complex, is cleaved by EV-D68 to inhibit host cell translation, while overexpression of PABPC1 enhances translation by increasing components of the translation initiation complex, thereby inhibiting EV-D68 replication. This mechanism is relatively straightforward. However, LARP1 is not a component of the translation initiation complex; instead, it regulates the initiation and shutdown of translation of specific mRNAs depending on cellular nutritional status. The mechanism by which LARP1 functions during EV-D68 infection requires further investigation. Here, we constructed truncated versions of LARP1 functional domains to study how LARP1 inhibits viral replication (Fig 3I) [55]. Both truncated and wild-type proteins were co-transfected into cells and infected with EV-D68 to determine their antiviral effects. The results showed that, compared to wild-type LARP1, some truncation mutants exhibited a slight compensatory inhibitory effect on viral replication, whereas the LAM domain truncation showed the most significant loss of suppression, indicating its critical role in LARP1-mediated antiviral activity (Fig 3J). The LAM domain is highly conserved across all LARPs and is usually accompanied by a downstream RNA recognition motif (RRM) [56]. This tandem arrangement forms the La-module [57]. Previous studies have reported that the LAM domain is involved in RNA binding [58,59], leading us to speculate that LARP1 may also interact with EV-D68 mRNA, further inhibiting EV-D68 replication.

### EV-D68 3C^pro^ cleaves LARP1 at Q371 disrupting its LAM domain and weakening its interaction with the viral 5’UTR

**Fig 4:**
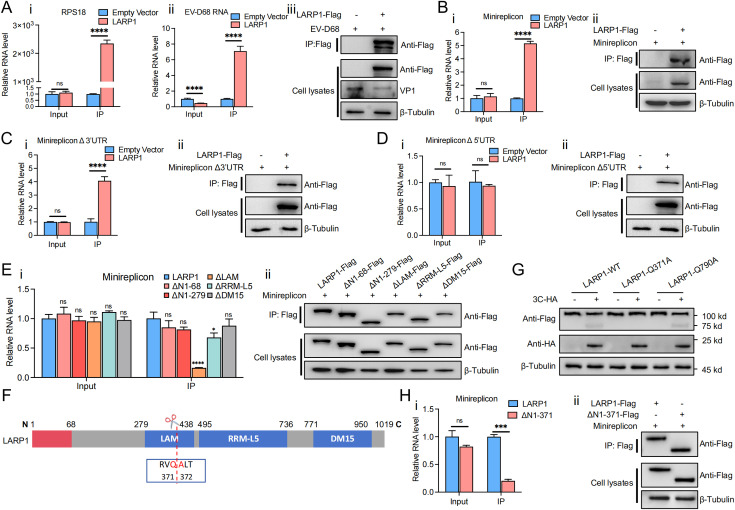
EV-D68 3C^pro^ cleaves LARP1 at Q371 disrupting its LAM domain and weakening its interaction with the viral 5’UTR. (A) LARP1-Flag plasmid was transfected into HEK293T cells for 24 h. Then HEK293T cells were infected with EV-D68 (MOI = 0.1) for 12 h. Cells were then collected for RIP, followed by qPCR (i, ii) and western blot(iii). (B - D) 293T cells were co-transfected with LARP1-Flag plasmid with EV-D68 minireplicon (B), EV-D68 minireplicon Δ3′UTR (C), EV-D68 minireplicon Δ5′UTR (D) for 36 h, respectively, and then the cells were collected for RIP, followed by qPCR(i) and western blot(ii). (E) HEK293T cells were co-transfected with various LARP1 deletion mutants (ΔN1-68, ΔN1-279, ΔLAM, ΔRRM-L5 and ΔDM15) along with the EV-D68 minireplicon for 36h. Cells were collected for RIP, followed by qPCR (i) and western blot analysis (ii). (F) Diagram of the functional domains and identified cleavage site of LARP1. (G) RD cells were co-transfected with LARP1-FLAG wild-type (WT), LARP1-Q371A-FLAG, or LARP1-Q790A-FLAG plasmids along with 3C-HA for 24 h. Cells were collected and subjected to western blot analysis. (H) HEK293T cells were transfected with full-length LARP1-FLAG or the ΔN1-371-FLAG for 36 h. Cells were then collected for RIP, followed by qPCR (i) and western blot (ii).

Studies have shown that LARP1 can interact with the 5’UTR of host mRNA [16,60]. To determine whether LARP1 interacts with EV-D68 RNA during infection, we transfected HEK293T cells with LARP1-Flag plasmid, infected them with EV-D68, and performed RNA immunoprecipitation (RIP) to detect RNA binding efficiency (Fig 4A). We also examined the expression of LARP1-Flag in cells before and after immunoprecipitation to confirm that LARP1-Flag was successfully enriched by Flag beads in the IP group. Meanwhile, we measured viral RNA and RPS18 mRNA levels before and after immunoprecipitation, as RPS18 mRNA has been reported to interact with LARP1 [61]. Surprisingly, the results showed that as LARP1 was purified and enriched, RPS18 mRNA levels were significantly enriched, while EV-D68 RNA levels were approximately 7-fold higher in the experimental group compared to the control group, confirming an interaction between LARP1 and EV-D68 RNA (Fig 4A).

To further investigate which part of EV-D68 RNA interacts with LARP1, we co-transfected HEK293T cells with LARP1 and minireplicons, followed by RIP to assess the interaction (Fig 4B-D). Immunoblotting analysis confirmed the expression and enrichment of LARP1-Flag in the IP group. qPCR analysis showed that, in the IP group, the full-length minireplicon RNA was enriched by approximately five-fold compared to the control group, indicating that LARP1 interacts with the EV-D68 minireplicon (Fig 4B). Further analysis showed that the Δ3’UTR minireplicon was enriched by approximately four-fold in the IP group compared to the control (Fig 4C). In contrast, the Δ5’UTR minireplicon was not enriched, and its mRNA levels were not significantly different from those in the control group, suggesting that the Δ5’UTR minireplicon lost its ability to interact with LARP1, indicating that LARP1 interacts with the 5’UTR of EV-D68 RNA (Fig 4D).

To further define the structural requirements for LARP1 interaction with EV-D68 RNA, we performed RIP assays with LARP1 truncations using EV-D68 minireplicon. RIP analysis showed that all LARP1 truncations were expressed at comparable levels in the input samples (Fig 4E, ii). However, deletion of the LAM domain (ΔLAM) significantly reduced LARP1’s binding ability to the EV-D68 minireplicon RNA. Additionally, deletion of RRM-L5 (ΔRRM-L5) resulted in a modest but statistically significant reduction in RNA binding, whereas other deletions (ΔN1–68, ΔN1–279, ΔDM15) had no significant effect. This suggests that the LAM domain is essential for LARP1’s interaction with EV-D68 RNA (Fig 4E).

We next investigated whether EV-D68 3C^pro^ cleavage directly impacts LARP1 function. Based on the observed endogenous LARP1 cleavage producing a 75 kDa fragment, the critical role of the LAM domain in EV-D68 RNA interaction, and previously reported EV-D68 3C^pro^ cleavage sequence logo analysis [62,63], we identified a similar cleavage motif within the LAM domain of LARP1, designating Q371 as a putative cleavage site (Fig 4F). To validate this, we generated the Q371A mutant and tested its cleavage sensitivity to 3C^pro^. To further assess the significance of this motif in LARP1’s sensitivity to 3C cleavage, we generated the Q790A mutant by mutating another potential cleavage site in the C-terminal region. Immunoblotting showed that wild-type LARP1 was cleaved by 3C^pro^, whereas the Q371A mutant was resistant, confirming that EV-D68 3C^pro^ cleaves LARP1 specifically at Q371. In contrast, the Q790A mutant did not exhibit significant resistance to cleavage, confirming that EV-D68 3C^pro^ cleaves LARP1 at Q371 (Fig 4G). To further confirm that the cleavage observed under overexpression of 3C^pro^ also occurs during EV-D68 infection, we performed cleavage assays with wild-type LARP1 and its cleavage site mutants Q371A and Q790A in the context of viral infection (S3 Fig). These results demonstrated that Q371A is resistant to cleavage, whereas Q790A does not confer protection, consistent with our observations using exogenous 3C^pro^.

Finally, we investigated whether LARP1 cleavage product affects its ability to bind EV-D68 RNA. To mimic the natural cleavage product of LARP1 generated during EV-D68 infection, we constructed the ΔN1–371 truncation mutant, retaining the C-terminal portion (N372-1019). RIP assays were then performed using full-length LARP1 and ΔN1–371 to assess RNA binding efficiency (Fig 4H). qPCR analysis showed that the ΔN1–371 mutant had significantly reduced binding to EV-D68 minireplicon RNA compared to full-length LARP1, confirming that cleavage at Q371 disrupts the interaction between LARP1 and the EV-D68 5’UTR (Fig 4H). These findings demonstrate that EV-D68 3C^pro^ cleaves LARP1 at residue Q371, leading to disruption of the LAM domain and weakening its interaction with the viral 5’UTR.

### EV-D68 infection impairs mTOR and CDK1 signaling pathways, leading to enhanced LARP1 binding to viral RNA

Available studies have shown that mTOR and CDK1 can regulate LARP1-mediated translation. LARP1 is a direct substrate of mTOR: non-phosphorylated LARP1 binds to the 5’ end of mRNA and inhibits translation. When mTOR is phosphorylated, LARP1 is also phosphorylated and activated, causing it to dissociate from the 5’ end of mRNA and promote translation [16,64,65]. Additionally, LARP1 activation is regulated by CDK1, a central hub connecting proliferation and mRNA translation. CDK1 and mTOR share common targets, including 4EBP1 and LARP1 [21]. Building upon our previous findings, we demonstrated that LARP1 interacts with the 5’UTR of EV-D68 RNA, with its LAM domain playing a critical role in this interaction. Prior studies have also shown that EV-D68 3C^pro^ cleaves LARP1 at the LAM domain, disrupting its ability to bind the 5’UTR of viral RNA. While LARP1 has been found to possess antiviral activity, truncation of its LAM domain weakens this effect. However, the precise relationship between LARP1’s antiviral role and its ability to bind viral RNA remains unclear. To further elucidate the regulatory mechanisms underlying LARP1-mediated antiviral activity, we investigated how mTOR and CDK1 signaling pathways contribute to this process.

**Fig 5:**
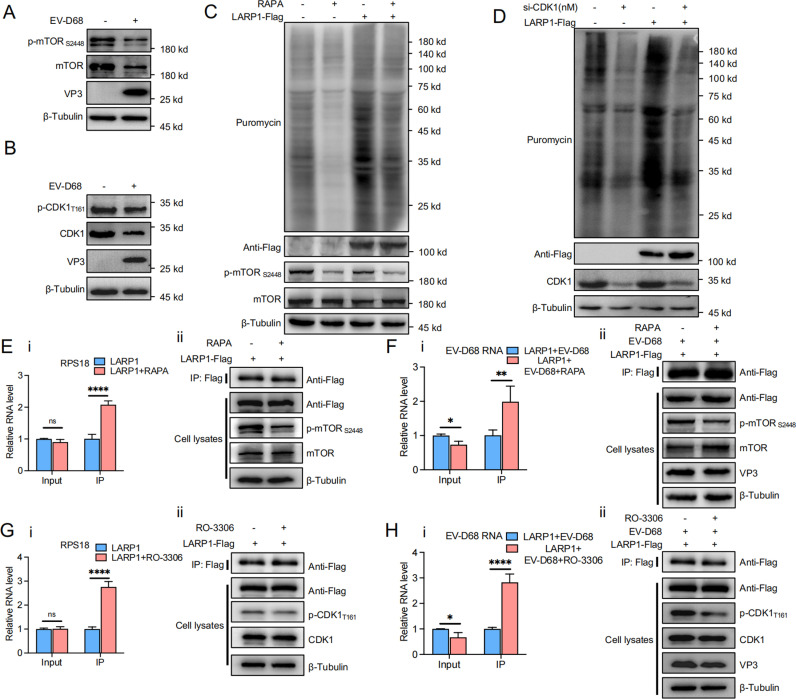
EV-D68 Infection Impairs mTOR and CDK1 signaling pathways, Leading to Enhanced LARP1 Binding to Viral RNA. (A, B) RD cells were infected with EV-D68 for 24 h and then cells were collected for western blot. (C) LARP1-Flag plasmid was transfected in RD cells for 24 h, then RAPA (10 µ M) was added and treated for 12 h. Cells were labelled with 10 µg/mL puromycin for 0.5 h, and then collected for western blot. (D) NC or pooled CDK1 siRNA was transfected in RD cells for 24 h, followed by transfection of the LARP1-Flag plasmid for 24 h. Cells were labelled with 10 µg/mL puromycin for 0.5 h, and then collected for western blot. (E) LARP1-Flag plasmid was transfected in HEK293T cells for 24 h, then RAPA (10 µ M) was added and treated for 12 h. Cell samples were collected for RIP, followed by qPCR(i) and western blot(ii). (F) HEK293T cells were transfected with the LARP1-Flag plasmid for 24 h, then infected with EV-D68. RAPA (10 µ M) was added 12 h post-infection, followed by cell collection for RIP analysis. Viral RNA levels were quantified by qPCR (i), and associated protein expression were validated by western blot (ii). (G) LARP1-Flag plasmid was transfected in HEK293T cells for 24 h, then RO-3306 (10 µ M) was added and treated for 12 h. Cell samples were collected for RIP, followed by qPCR(i) and western blot(ii). (H) HEK293T cells were transfected with the LARP1-Flag plasmid for 24 h, then infected with EV-D68. RO-3306 (10 µ M) was added 12 h post-infection, followed by cell collection for RIP analysis. Viral RNA levels were quantified by qPCR (i), and associated protein expression were validated by western blot (ii).

First, we infected RD cells with EV-D68 to assess the activation of the mTOR signaling pathway. Immunoblotting revealed that mTOR and p-mTOR_S2448_ levels were significantly reduced upon EV-D68 infection, suggesting that EV-D68 impairs mTOR signaling, which may contribute to increased LARP1 binding to viral RNA while potentially affecting LARP1 activation (Fig 5A). We also analyzed CDK1 expression and its phosphorylated form (p-CDK1_T161_), indicating decreased CDK1 activation and suggesting that CDK1 plays a regulatory role in LARP1-mediated translation and RNA binding (Fig 5B).

To confirm mTOR’s effect on LARP1, we used Rapamycin (RAPA), a known mTOR inhibitor [66]. After transfecting LARP1-Flag plasmid, cells were treated with RAPA (10 µ M) and then collected for immunoblotting. The results showed that RAPA-mediated inhibition of mTOR phosphorylation led to a decrease in LARP1-driven mRNA translation, indicating that mTOR plays a regulatory role in LARP1-mediated translational control (Fig 5C). As an additional validation of mTOR signaling pathway involvement, we performed the same set of experiments using the mTOR inhibitor Torin1, which is known for its broader inhibition of both mTORC1 and mTORC2 (S4A Fig). Torin1 treatment similarly suppressed LARP1-driven mRNA translation, consistent with the effects observed under RAPA treatment. To investigate the role of CDK1 in LARP1 regulation, we performed CDK1 knockdown using CDK1 siRNA. The results demonstrated that CDK1 depletion led to a significant reduction in LARP1-induced mRNA translation, suggesting that CDK1 is an important regulator of LARP1-mediated translational activity (Fig 5D). These findings further support the role of mTOR and CDK1 as upstream regulators of LARP1, influencing its function in translational regulation, as indicated by our previous analysis of LARP1 truncations and cleavage by EV-D68 3C^pro^. Since EV-D68-induced LARP1 cleavage disrupts its function, we next explored whether EV-D68-mediated inhibition of mTOR and CDK1 signaling pathways further impacts LARP1 activity, particularly in its ability to bind viral RNA.

HEK293T cells were transfected with LARP1-Flag plasmid, treated with RAPA, and then subjected to RIP. The results showed that LARP1-Flag enrichment by Flag beads in the RAPA-treated group led to significantly higher RPS18 mRNA levels compared to the control, indicating that mTOR inhibition enhances LARP1-RNA interaction, preventing LARP1’s normal dissociation from RNA and inhibiting translation (Fig 5E). In addition, we used the same experimental setup to assess the effects of RAPA treatment on LARP1 binding during EV-D68 infection. The results demonstrated that the RAPA-treated group had significantly higher levels of EV-D68 RNA co-purified with LARP1 compared to the control, suggesting that mTOR inhibition enhances LARP1-EV-D68 RNA interaction, impairing translation of EV-D68 RNA (Fig 5F). RO-3306 is a CDK1 inhibitor [67]. To further explore the impact of CDK1 on LARP1 function, we repeated the experiment using RO-3306. The results showed that RO-3306 treatment led to significantly higher RPS18 mRNA levels co-purified with LARP1 compared to the control, indicating that CDK1 inhibition enhances LARP1-RNA interaction, preventing LARP1’s normal dissociation from RNA and inhibiting translation (Fig 5G). The results also demonstrated that RO-3306 treatment led to significantly higher levels of EV-D68 RNA co-purified with LARP1 compared to the control, suggesting that CDK1 inhibition enhances LARP1-EV-D68 RNA interaction, reducing EV-D68 RNA translation (Fig 5H). Additionally, we performed densitometric analysis of p-mTOR/mTOR and p-CDK1/CDK1 intensity band ratios, and the results are presented in S4B-E Fig.

Together, these findings establish a mechanistic link between mTOR/CDK1 signaling pathways and LARP1 function, building on our observations in Fig 4 to further investigate how these pathways modulate LARP1 activity and its interaction with viral RNA. Under normal conditions, mTOR and CDK1 signaling pathways promote LARP1 phosphorylation, facilitating its dissociation from bound RNA and enhancing translation efficiency. During EV-D68 infection, mTOR and CDK1 signaling pathways are inhibited, leading to impaired activation of LARP1, enhancing its binding to the viral 5’UTR through its LAM domain, thereby suppressing viral RNA translation. To circumvent LARP1-mediated suppression of its own translation, EV-D68 3C^pro^ cleaves the LAM domain of LARP1, leading to the release of viral RNA from LARP1-mediated suppression, thereby facilitating viral RNA translation.

### Potential universal inhibitory role of PABPC1 and LARP1 in enteroviruses

**Fig 6:**
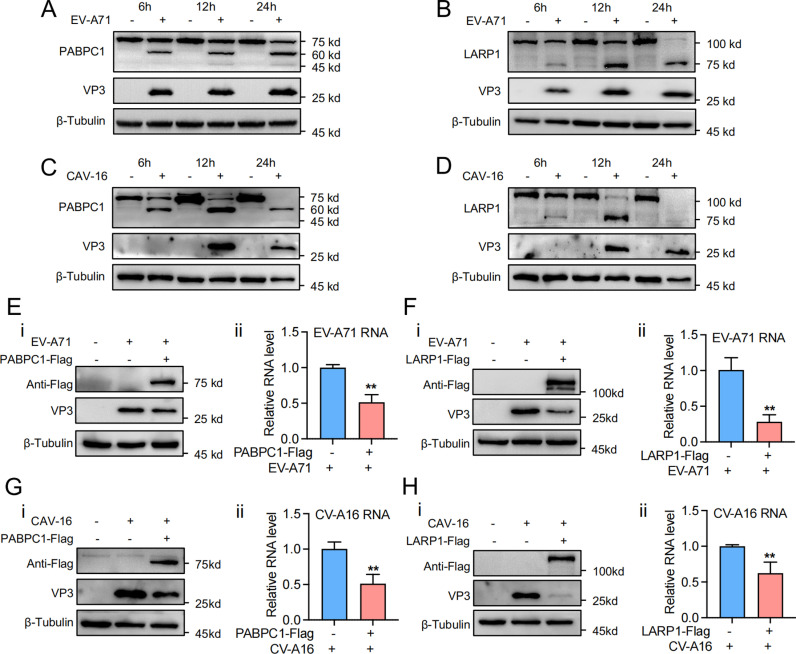
Potential Universal Inhibitory Role of PABPC1 and LARP1 in Enteroviruses. (A, B) RD cells were infected with EV-A71 (MOI = 0.1) for 6 h, 12 h and 24 h, Cells were collected for western blot. (C, D) RD cells were infected with CV-A16 (MOI = 0.5), and cells infected for 6 h, 12 h and 24 h were collected for western blot. (E) PABPC1-Flag plasmid was transfected in RD cells for 24 h, then infected with EV-A71 (MOI = 0.1), and the lysates of the cells were collected after 24 h for western blot (i), qPCR (ii). (F) LARP1-Flag plasmid was transfected in RD cells for 24 h, then infected with EV-A71 (MOI = 0.1), and the lysates of the cells were collected after 24 h for western blot (i), qPCR (ii). (G) PABPC1-Flag plasmid was transfected in RD cells for 24 h, then infected with CV-A16 (MOI = 0.5), and the lysates of the cells were collected after 24 h for western blot (i), qPCR (ii). (H) LARP1-Flag plasmid was transfected in RD cells for 24 h, then infected with CV-A16 (MOI = 0.5), and the lysates of the cells were collected after 24 h for western blot (i), qPCR (ii).

To determine whether the mechanisms observed in EV-D68 apply to other enteroviruses, we extended our study to EV-A71 and CV-A16. We first investigated changes in PABPC1 and LARP1 during EV-A71 and CV-A16 infection over time. RD cells were infected with EV-A71 and collected at 6, 12, and 24 hours post-infection for immunoblotting. The results showed that EV-A71 progressively cleaved PABPC1, with distinct cleavage bands appearing at 60 kDa and 45 kDa (Fig 6A). Similarly, LARP1 was cleaved over time, with a prominent band at 75 kDa (Fig 6B). We conducted a similar experiment with CV-A16 under the same conditions. CV-A16 also cleaved PABPC1 and LARP1 over time, showing similar patterns to those observed with EV-A71 (Fig 6C, D). These findings indicate that EV-A71 and CV-A16 target PABPC1 and LARP1 for cleavage during infection.

To explore the effects of PABPC1 and LARP1 on viral replication, we overexpressed PABPC1-Flag or LARP1-Flag in RD cells, followed by infection with EV-A71 or CV-A16. The cells were collected for immunoblotting and qPCR. Overexpression of PABPC1 or LARP1 significantly reduced EV-A71 protein and RNA levels by approximately 50% and 60%, respectively (Fig 6E, F). Similar inhibitory effects were observed with CV-A16, where overexpression of either protein suppressed viral protein and RNA levels (Fig 6G, H). These results suggest that PABPC1 and LARP1 inhibit EV-A71 and CV-A16 replication.

Overall, these findings indicate that EV-A71 and CV-A16 use a strategy similar to EV-D68, targeting PABPC1 and LARP1 for cleavage to escape inhibition and promote replication. The suppression of viral replication upon overexpression of these proteins suggests a potential universal inhibitory role of PABPC1 and LARP1 in enteroviruses, indicating that these proteins are key targets in the viral strategy to evade host defenses.

## Discussion

EV-D68, known for causing respiratory diseases, viral meningitis, and acute flaccid myelitis, poses a significant threat to global public health. Previous studies have shown that EV-D68 hijacks host translation machinery by cleaving eIF4G through its 2A^pro^, making host cap-dependent translation ineffective while facilitating viral IRES-dependent translation initiation [42–44]. In this study, we elucidated the intricate relationship between EV-D68 and host mRNA translation, highlighting how EV-D68 hijacks host translation machinery to promote its replication. Our findings build on previous knowledge, adding new layers of understanding to the viral manipulation of host translation.

Our study demonstrated that during EV-D68 infection, the viral 3C^pro^ cleaves LARP1, while PABPC1 is cleaved by both 2A^pro^ and 3C^pro^. This cleavage disrupts the interaction between cap-binding protein eIF4E and mRNA, impairing the recruitment of the translation initiation complex, which ultimately suppresses host translation. This disruption of normal translation initiation represents a significant viral strategy to divert cellular resources away from host protein production. Unlike host mRNA, EV-D68 RNA translation does not require eIF4E, as it can directly recruit the scaffold protein eIF4G, making the translation machinery more accessible for viral replication [30].

Interestingly, we found that overexpression of PABPC1 and LARP1 in RD cells inhibits EV-D68 replication and attenuates the virus-induced suppression of host translation. While our findings do not suggest a direct cooperative effect between PABPC1 and LARP1, we observed that knocking down PABPC1 reduced exogenous LARP1 expression. This may be due to PABPC1’s role in translation complex assembly, where its depletion could indirectly affect transient plasmid expression efficiency, leading to a decrease in exogenous LARP1 levels. PABPC1, as a component of the translation initiation complex, enhances host translation by increasing the availability of components for the initiation complex, thereby reducing the effectiveness of EV-D68’s hijacking strategy. LARP1, however, is not part of the translation initiation complex; it regulates initiation or shutdown of specific mRNA translation based on cellular nutritional status. So, what is the specific mechanism by which LARP1 inhibits EV-D68 replication?

To further investigate the functional mechanism of LARP1, we constructed truncated versions of its functional domains and found that truncation of the LAM domain significantly weakened LARP1’s antiviral activity. Additionally, ΔN1–68, ΔN1–279, ΔRRM-L5, and ΔDM15 exhibited some compensatory effects, but these effects were relatively weak. Since protein domains often function interdependently, removing one region may alter the stability and function of adjacent domains, further contributing to LARP1 dysfunction. The truncations △1–68 and △1–279 may disrupt LARP1’s structural integrity, thereby impairing its ability to regulate translation. Similarly, DM15 located at the C-terminus of LARP1, plays a key role in binding transcripts with 7-methylguanosine (m7G) caps and 5′TOP motifs [68], and its truncation may impair these interactions, affecting LARP1’s regulatory role in translation. Furthermore, disrupting DM15 may reduce LARP1’s RNA-binding capacity, which could contribute to the observed decrease in antiviral efficacy. The LAM domain typically collaborates with the RRM domain to form an RNA scaffold, known as the La-module [57], and truncation of either domain weakened the viral inhibitory effect, with the LAM domain truncation exhibiting the weakest inhibition. RRM-L5 participates in RNA recognition within the La-module, and its truncation may impair LARP1’s ability to recognize RNA, potentially weakening its antiviral function. Given that both the LAM domain and RRM-L5 contribute to RNA binding, we hypothesized that LARP1 may directly interact with viral RNA, influencing its translation.

To investigate this hypothesis, we examined the RNA-binding capability of LARP1 and its potential interaction with viral RNA. As expected, we found that LARP1 interacts with the 5’UTR of EV-D68 RNA, with its LAM domain playing a crucial role in this interaction. Meanwhile, we also considered the role of RRM-L5 in the La-module and its involvement in RNA recognition. In RIP assays, RRM-L5 truncation indeed reduced its ability to associate with viral RNA compared to wild-type LARP1. However, surprisingly, its binding capacity was higher than that of the LAM domain truncation, raising the question of whether the LAM domain alone could effectively bind RNA. Supporting our speculation, Jiang et al. previously demonstrated that the LAM domain alone is sufficient for RNA binding [69]. This suggests that while RRM-L5 contributes to RNA recognition, the LAM domain is the primary determinant for viral RNA binding and translational regulation. Moreover, our results indicate that EV-D68 3C^pro^-mediated cleavage of LARP1 occurs specifically at residue Q371, disrupting its interaction with viral RNA. Compared to wild-type LARP1, the cleavage product lost its ability to bind viral RNA. Given this loss of function, we hypothesized that LARP1’s RNA-binding activity might be dynamically regulated by host signaling pathways, influencing its role in translation. Subsequently, we further investigated the functional role of LARP1.

Previous studies have shown that LARP1 activity is linked to mTOR and CDK1 signaling pathways, both of which play crucial roles in translation regulation [16,21,70,71]. We observed that viral infection inhibits these pathways, thereby impacting LARP1’s ability to regulate translation. This suggests that LARP1’s modulation of translation is intricately linked to these pathways and may serve as a regulatory mechanism through which host signaling influences viral replication dynamics. To validate this, we utilized mTOR and CDK1 inhibitors, which mimicked the effects of viral infection, resulting in increased LARP1 binding to viral RNA. These findings suggest that host signaling pathways modulate LARP1’s function as a translation regulator, adding another layer of complexity to its role during EV-D68 infection. It is well established that enteroviruses induce autophagy in the later stages of infection to facilitate the release of newly replicated viral particles [72,73]. Many studies, particularly on EV-A71, have shown that enteroviruses suppress mTOR activity to promote autophagy, thereby enhancing viral replication [74,75]. Thus, the inhibition of mTOR observed in our study is likely a viral strategy to stimulate autophagy and facilitate viral release. Regarding CDK1, studies have shown that CDK1 phosphorylates key autophagy-related proteins, including ULK1, ATG13, and Vps34, thereby suppressing the autophagic process [76,77]. Consequently, inhibition of CDK1 activity may release this suppression, leading to enhanced autophagy. Interestingly, while the virus suppresses mTOR and CDK1 to promote autophagy and facilitate viral particle release, this same process enhances LARP1’s binding to viral RNA, restricting viral translation. This regulatory mechanism forms a feedback loop where the virus exploits host autophagy to maximize replication, but LARP1 responds to these host-induced changes to counteract viral replication. To break this feedback loop, EV-D68 3C^pro^ cleaves LARP1 at residue Q371, disrupting this interaction and releasing viral RNA, thereby promoting efficient viral gene expression. Cleavage of LARP1 disrupts its translation-inhibitory effects and correlates with increased viral replication. This targeted cleavage serves as an adaptive mechanism to bypass host-imposed translational barriers, ensuring a competitive advantage in viral gene expression. This also explains why EV-D68 cleaves LARP1 during infection.

**Fig 7:**
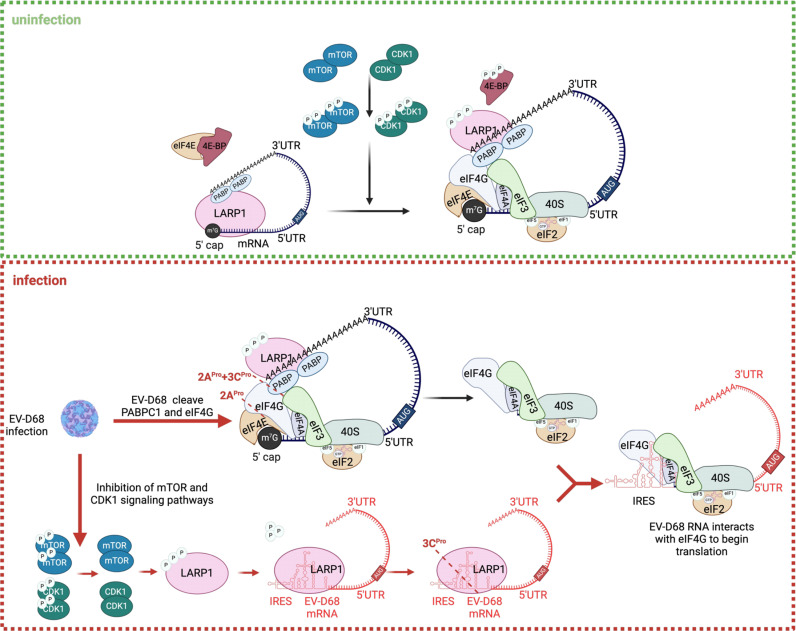
Proposed model for EV-D68 cleavage of LARP1 and PABPC1 to inhibit host cell translation and redirect translation initiation factors for viral RNA translation. Under normal nutrient available conditions, mTOR and CDK1 regulate the dissociation of downstream LARP1 from bound mRNA through phosphorylation, allowing the eIF4E protein to bind to the 5′ end of the mRNA, thereby recruiting the translation initiation complex and initiating translation. EV-D68 infection reduces the protein levels of mTOR and CDK1 in the cell, and the corresponding phosphorylation levels are also reduced, resulting in the inability of LARP1 to dissociate from the binding RNA, and EV-D68 translation is inhibited. To this end, EV-D68 cleaves LARP1 and PABPC1 so that the translation initiation site of viral RNA is not occupied by LARP1 and is able to bind to translation initiation factors and recruit the corresponding translation initiation proteins for its own use. Created in BioRender. Ruyang, T. (2025) https://BioRender.com/c23t611.

The mechanism diagram further illustrates the interplay between EV-D68 and host translation machinery (Fig 7). Under normal conditions, mTOR and CDK1 are phosphorylated and allowing LARP1 to dissociate from the 5’ cap of bound host mRNA (blue-marked). This dissociation permits recruitment of the translation initiation complex to initiate translation. Upon EV-D68 infection, activation of mTOR and CDK1 pathways is inhibited, preventing LARP1 from dissociating from bound viral mRNA (red-marked), thereby impairing translation initiation of viral mRNAs. To overcome this inhibition, EV-D68 3C^pro^ cleaves LARP1, freeing the bound viral mRNA to initiate translation. Concurrently, EV-D68 infection cleaves eIF4G and PABPC1, inhibiting cap-dependent host translation and commandeering the liberated initiation factors for viral replication. Thus, by cleaving PABPC1 and LARP1, the viral mRNAs are prioritized for translation over host mRNAs, optimizing the recruitment of translation initiation factors to support efficient viral replication. This highlights how EV-D68 fine-tunes host translational control to maximize its own gene expression, underscoring the intricate virus-host interactions at play.

In summary, EV-D68 employs a sophisticated strategy to hijack host translation machinery by selectively cleaving translation-related proteins like PABPC1 and LARP1, thereby inhibiting host mRNA translation and redirecting the translation machinery for viral use. Moreover, this mechanism, which may be conserved among enteroviruses, provides new insights into viral-host interactions, and identifies potential targets for antiviral strategies.

## Materials and methods

### Cell lines and viruses

Rhabdomyosarcoma (RD) cells (CRL-136; American Type Culture Collection [ATCC], Manassas, VA, USA), human embryonic kidney 293T (HEK293T) cells (CRL-11268; ATCC), and HeLa cells (CCL-2; ATCC) were cultured in Dulbecco’s modified Eagle’s medium (DMEM) supplemented with 10% fetal bovine serum (FBS) and 1% penicillin-streptomycin (100 IU/ml) at 37°C in a 5% CO_2_ humidified atmosphere. The EV-D68 Fermon strain (GenBank accession no. KU844179.1) was generously provided by Professor Xiaofang (Department of Molecular Microbiology and Immunology, Johns Hopkins Bloomberg School of Public Health, Baltimore, MD, USA). The EV-A71 strain was provided by Professor Fei Guo (Chinese Academy of Medical Sciences). The CV-A16 strain was provided by Professor Yaxin Wang (College of Life Sciences, Tianjin University).

### Antibodies and reagents

LARP1 Polyclonal antibody (13708–1-AP), EIF4E Polyclonal Antibody (11149–1-AP), MTOR Polyclonal Antibody (28273–1-AP), CDK1 Polyclonal Antibody (19532–1-AP), anti-tubulin Monoclonal antibody (66031–1-Ig), and DYKDDDDK Tag Polyclonal Antibody (Binds To FLAG Tag Epitope) (20543–1-AP) were purchased from Proteintech Group. β-Actin Monoclonal antibody (AC026), HA-Tag Monoclonal antibody (AE008), HA-Tag Polyclonal antibody (AE036), and Myc-Tag Monoclonal antibody (AE070), PABPC1 Polyclonal antibody (A24646), Puromycin Polyclonal antibody (A21205), Phospho-mTOR-S24489 Monoclonal antibody (AP0115) were purchased from ABclonal Technology. GAPDH Mouse mAb (KM9002) were purchased from Sungene Biotech. EV-D68 VP3 Polyclonal antibody (GTX132315) were purchased from GeneTex. The secondary antibodies including goat anti-rabbit horseradish peroxidase (HRP)-conjugated IgG(H + L) antibody (LK2001; Sungene Biotech), and HRP-conjugated anti-mouse IgG antibody (SA00001–1; Proteintech). The enhanced chemiluminescence (ECL) reagent was obtained from Thermo Fisher Scientific.

4’, 6-diamidino-2-phenylindole (DAPI; H1200), and Rapamycin (RAPA) (R8140) was acquired from Solarbio Life Science. TransScript II First-Strand cDNA Synthesis SuperMix (AH301–02), FastPfu DNA polymerase (M21105), TransDetect Double-Luciferase Reporter Assay Kit (FR201–01), and TransStart Top Green qPCR SuperMix (AQ131–01) were purchased from TransGen (Beijing, China). Anti-Flag M2 Affinity Gel (A2220), Duolink In Situ Detection Reagents Red (DUO92002), Duolink In Situ PLA Probe Anti-Mouse PLUS(DUO92001) and Duolink In Situ PLA Probe Anti-Mouse MINUS (DUO92006) were purchased from Sigma. Anti-HA affinity matrix (Product No.11815016001) was purchased from Roche.

### Plasmids

VR1012 plasmid was donated by Professor Zhang Wenxue (Jilin University). The pcDNA3.1 plasmid was stored in our laboratory. Myc-LARP1-Flag plasmid was purchased from Wuhan Miaoling Biology (P43704). The plasmid expressing PABPC1-FLAG, EIF4G-FLAG, and 4EBP1-HA plasmid variants was generated by PCR amplification from HEK293T cDNA and cloned into VR1012 and pcDNA3.1 vector.

PABPC1-FLAG (F:5’GACACGTGTGATCAGATATCATGAACCCCAGTGCCCCCAG3’, R:5’GATCCAGGGCCTGGTCTAGATTACTTGTCATCGTCGTCCTTGTAATCAACAGTTGGAACACCGGTGGC3’); EIF4G-Flag (F:5’CGACACGTGTGATCAGATATCATGAACAAAGCTCCACAGTCCACAGG3’, R:5’GGATCCAGGGCCTGGTCTAGATTACTTGTCATCGTCGTCCTTGTATCGTTGTGGTCAGACTCCTCCTCTGC3’); 4EBP1-HA (F:5’AGTCCAGTGTGGTGGAATTCGATGTCCGGGGGCAGCAG3’, R:5’ AACGGGCCCTCTAGACTCGAGTTAAATGTCCATCTCAAACTGTGACTCTTCACC3’).

### RNA extraction and qPCR

Total cellular RNA was extracted by TRIzol (Qiagen, Germany) extraction. Using TransScript II First-Strand cDNA Synthesis SuperMix to reverse transcription of RNA to cDNA with oligo(dT) primers. RT-qPCR assay was performed using TransStart Top Green qPCR SuperMix, and mRNA levels were normalized to GAPDH mRNA. Results were analyzed as fold change using the 2-ΔΔCt method.

Primers were used for EV-D68 mRNA (F:5’GGCAGCCTATCAGGTGGAGAG3’, R:5’GAGTTTGTATGGCTTCTTCTGGT3’); EV-A71 mRNA (F:5’GTCCTTAATTCGCACAGCACAGCT3’, R:5’CGGTCCGCACTGAGAATGTACCCAT3’); RPS18 (F:5’TGTGGTGTTGAGGAAAGCAG3’, R:5’AAGTGACGCAGCCCTCTATG3’).

### RNA interference

Cells are first seeded in a 24-well plate one day before transfection to achieve 50–60% confluency the following day. At this density, siRNA transfection is initiated. Dilute 1 μL of Lipofectamine 2000 (Lipo2000) and the required concentration of siRNA in 50 μL of Opti-MEM separately and incubate at room temperature for 5 minutes. Combine the diluted Lipo2000 solution with the diluted siRNA solution, gently mix, and incubate at room temperature for 20 minutes. Replace the existing cell culture medium in each well with 100 μL of Opti-MEM, and add the transfection complex to each corresponding well, resulting in a total volume of 200 μL per well. After 4 hours of transfection, remove the transfection complex, and replace it with 10% complete medium or 2% maintenance medium, according to experimental requirements. Continue culturing the cells for an additional 48–72 hours.

### Coimmunoprecipitation and immunoblotting

Transfect the required plasmid into HEK293T cells. After 48 h, the cells were harvested and lysed with lysis buffer. The lysates were placed at 4°C for 30 min and transferred 1/10 as input lysates to detect the total cells expression. The remaining lysates was incubated with Anti-Flag M2 Affinity Gel or Anti-HA affinity matrix at 4°C for 6 h. The beads were washed with 1 mL of cold PBS for six times and eluted with a glycine elution buffer to get immunoprecipitates. The samples in SDS loading buffer were boiled and use Western blotting to detect protein expression. Western blotting was performed according to the conventional method as described previously [78].

### RNA immunoprecipitation (RIP)

Cells were resuspended in 2 mL PBS, with 200 μL transferred to a sterile 1.5 mL EP tube and 1.8 mL to a 2.0 mL EP tube, followed by centrifugation at 6500 rpm for 5 min. The pellet from the 200 μL suspension was stored at -80°C for RNA extraction (qPCR input). The 1.8 mL pellet was lysed with 700 μL lysis buffer, rotated at 4°C for 30 min, and centrifuged at 7000 rpm for 10 min. A 60 μL supernatant was taken for protein immunoblot analysis, mixed with 15 μL 5x loading buffer, and heated at 99°C for 10 min. The remaining lysate was incubated with HA/Flag beads at 4°C for 6 h, then centrifuged at 7000 rpm for 2 min, with washes using 500 μL PBS repeated 5–6 times on ice. The final supernatant was split into two parts: one stored at -80°C for RNA extraction (qPCR IP sample) and the other mixed with 40 μL glycine elution buffer, incubated for 5 min, and centrifuged. The resulting 40 μL supernatant was used as an IP sample for immunoblot, mixed with 10 μL 5x loading buffer, neutralized with 3 μL neutralization buffer until the color changed from yellow to blue, heated at 99°C for 10 min, and stored for analysis.

### Statistical analysis

Data were analyzed with GraphPad Prism and are expressed as mean and standard error. Statistical significance was determined using two-tailed Student’s unpaired t-test (*P < 0.05, **P < 0.01, ***P < 0.001).

## Supporting information

S1 Fig (A) HEK293T cells were transfected with PABPC1-Flag plasmid for 48 h. Cells were collected for Co-IP. (B) HeLa cells were transfected with LARP1-Flag plasmid for 48 h and the interaction between endogenous PABPC1 and LARP1-Flag was detected by Duolink assay. Scale bar = 10 μm; HeLa cells were transfected with PABPC1-Flag plasmid for 48 h and the interaction between endogenous LARP1 and PABPC1-Flag was detected by Duolink assay. Scale bar = 10 μm. (C) RD cells were infected with EV-D68 (MOI = 0.1), and cells infected for 6 h, 12 h and 24 h were collected for qPCR. (D) HEK293T cells were infected with EV-D68 (MOI = 0.1), and cells infected for 6 h, 12 h and 24 h were collected for qPCR (i) and western blot (ii), respectively.
(TIF)

S2 Fig (A) Gradient transfection of LARP1-Flag plasmid in RD cells for 24 h, followed by infection with EV-D68, after 12 h/24 h for bright-field observation by microscopy. Scale bar = 10 μm. (B) PABPC1 siRNA was transfected in RD cells for 24 h, then transfected with LARP1-Flag plasmid for 24 h, followed by infection with EV-D68 virus for 24 h. Finally, samples were collected for qPCR. (C) Empty Vector, PABPC1-Flag, and PABPC1-Flag were transfected in RD cells, PABPC1-Flag and EV-D68 minireplicon, transfected for 24 h, then infected with EV-D68, and cells were collected after 12 h for qPCR. (D) Empty Vector, LARP1-Flag, LARP1-Flag, and EV-D68 minireplicon were transfected in RD cells for 24 h, then infected with EV-D68, and the cells were collected for qPCR after 12 h.
(TIF)

S3 Fig RD cells were transfected with LARP1-FLAG wild-type (WT), LARP1-Q371A-FLAG, or LARP1-Q790A-FLAG plasmids for 24 h, then infected with EV-D68, and the lysates of the cells were collected after 24 h for western blot. (TIF)

S4 Fig (A) LARP1-Flag plasmid was transfected in RD cells for 24 h, then Torin1 (10 µ M) was added and treated for 12 h. Cells were labelled with 10 µg/mL puromycin for 0.5 h, and then collected for western blot. (B) HEK293T cells were transfected with LARP1-Flag plasmid for 24 h, followed by treatment with or without RAPA (10 µ M) for 12 h. The phosphorylation level of mTOR was analyzed by western blot, and the p-mTOR/mTOR band intensity ratio was quantified. (C) HEK293T cells were co-transfected with LARP1-Flag plasmid and infected with EV-D68 (MOI = 0.1) for 12 h, followed by treatment with or without RAPA (10 µ M) for 12 h. Western blot analysis was conducted to examine mTOR phosphorylation levels, and the p-mTOR/mTOR band intensity ratio was quantified. (D) HEK293T cells were transfected with LARP1-Flag plasmid for 24 h, followed by treatment with or without RO-3306 (10 µ M) for 12 h. Western blot analysis was performed to assess CDK1 phosphorylation levels, and the p-CDK1/CDK1 band intensity ratio was quantified. (E) HEK293T cells were co-transfected with LARP1-Flag plasmid and infected with EV-D68 (MOI = 0.1) for 12 h, followed by treatment with or without RO-3306 (10 µ M) for 12 h. The phosphorylation level of CDK1 was analyzed by western blot, and the p-CDK1/CDK1 band intensity ratio was quantified.
(TIF)

S1 Data siRNA Sequence. (XLSX)

S2 Data Source data for Fig 2D, 3A-3B, 3I, 4A-4E, 4H, 5E-5H, 6E-6H, sFig1C-1D, 2B-2D, 3A-3D. (XLSX)
