# Supplementary figures and images for: EV-D68 cleaves LARP1 and PABPC1 by 3Cpro to redirect host mRNA translation machinery toward its genomic RNA
Source: PLoS Pathog. 2025 Apr 28;21(4):e1013098. doi: 10.1371/journal.ppat.1013098 (PMC12036898; doi:10.1371/journal.ppat.1013098)

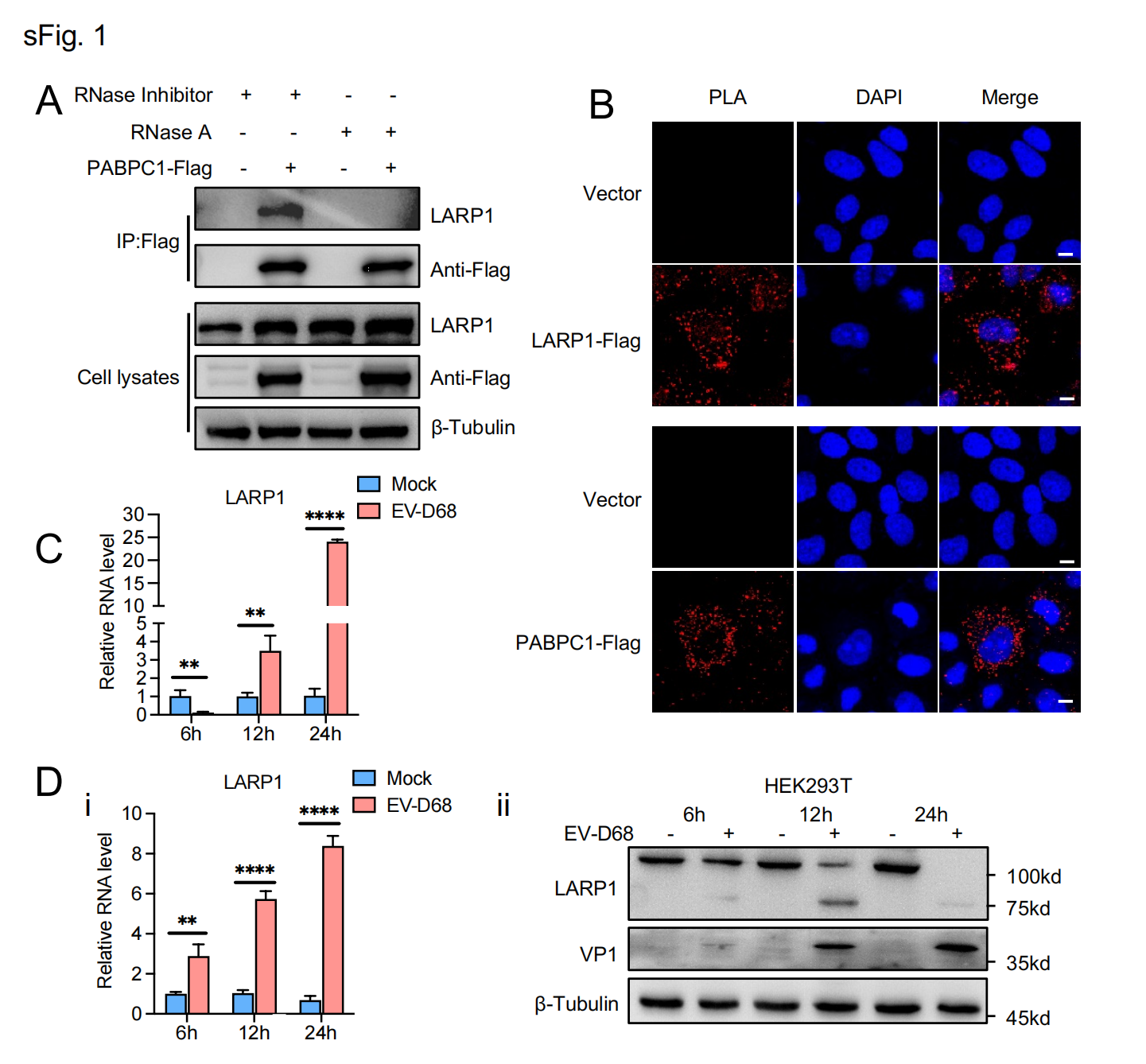

Supplement: S1 Fig — Cells were collected for Co-IP. (B) HeLa cells were transfected with LARP1-Flag plasmid for 48 h and the interaction between endogenous PABPC1 and LARP1-Flag was detected by Duolink assay. Scale bar = 10 μm; HeLa cells were transfected with PABPC1-Flag plasmid for 48 h and the interaction between endogenous LARP1 and PABPC1-Flag was detected by Duolink assay. Scale bar = 10 μm. (C) RD cells were infected with EV-D68 (MOI = 0.1), and cells infected for 6 h, 12 h and 24 h were collected for qPCR. (D) HEK293T cells were infected with EV-D68 (MOI = 0.1), and cells infected for 6 h, 12 h and 24 h were collected for qPCR (i) and western blot (ii), respectively. (TIF) [file ppat.1013098.s001.tif]

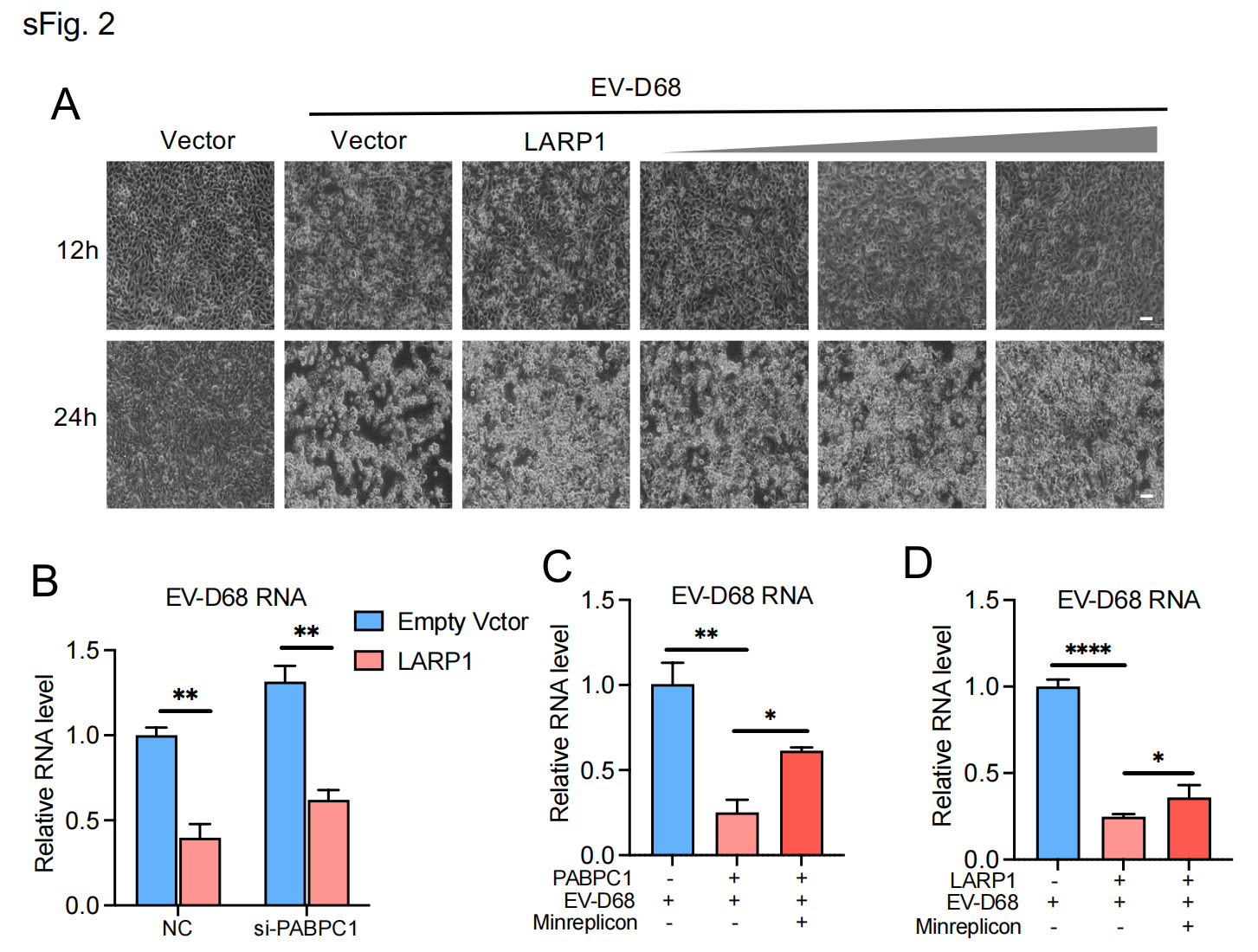

Supplement: S2 Fig — Scale bar = 10 μm. (B) PABPC1 siRNA was transfected in RD cells for 24 h, then transfected with LARP1-Flag plasmid for 24 h, followed by infection with EV-D68 virus for 24 h. Finally, samples were collected for qPCR. (C) Empty Vector, PABPC1-Flag, and PABPC1-Flag were transfected in RD cells, PABPC1-Flag and EV-D68 minireplicon, transfected for 24 h, then infected with EV-D68, and cells were collected after 12 h for qPCR. (D) Empty Vector, LARP1-Flag, LARP1-Flag, and EV-D68 minireplicon were transfected in RD cells for 24 h, then infected with EV-D68, and the cells were collected for qPCR after 12 h. (TIF) [file ppat.1013098.s002.tif]

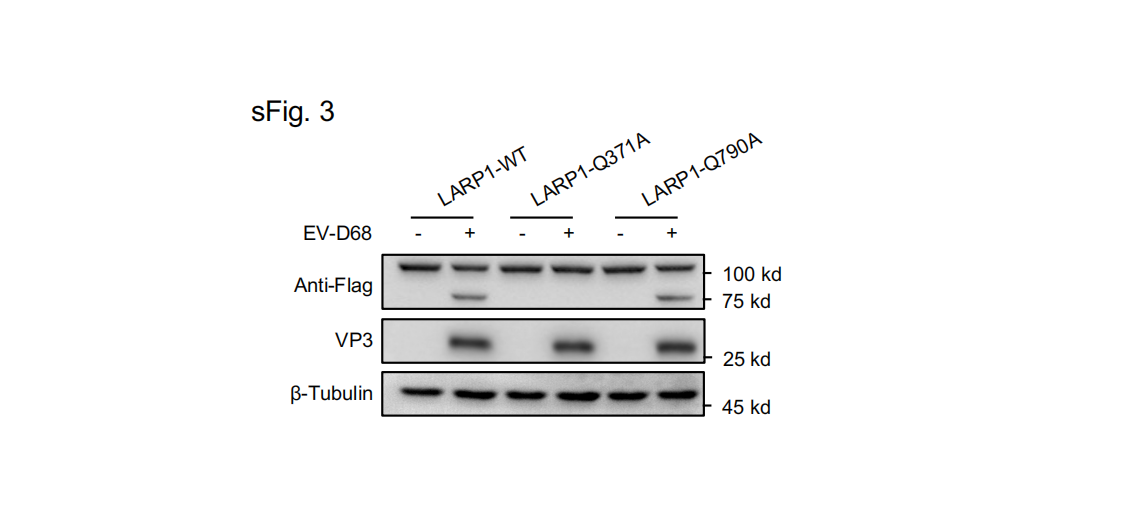

Supplement: S3 Fig — (TIF) [file ppat.1013098.s003.tif]

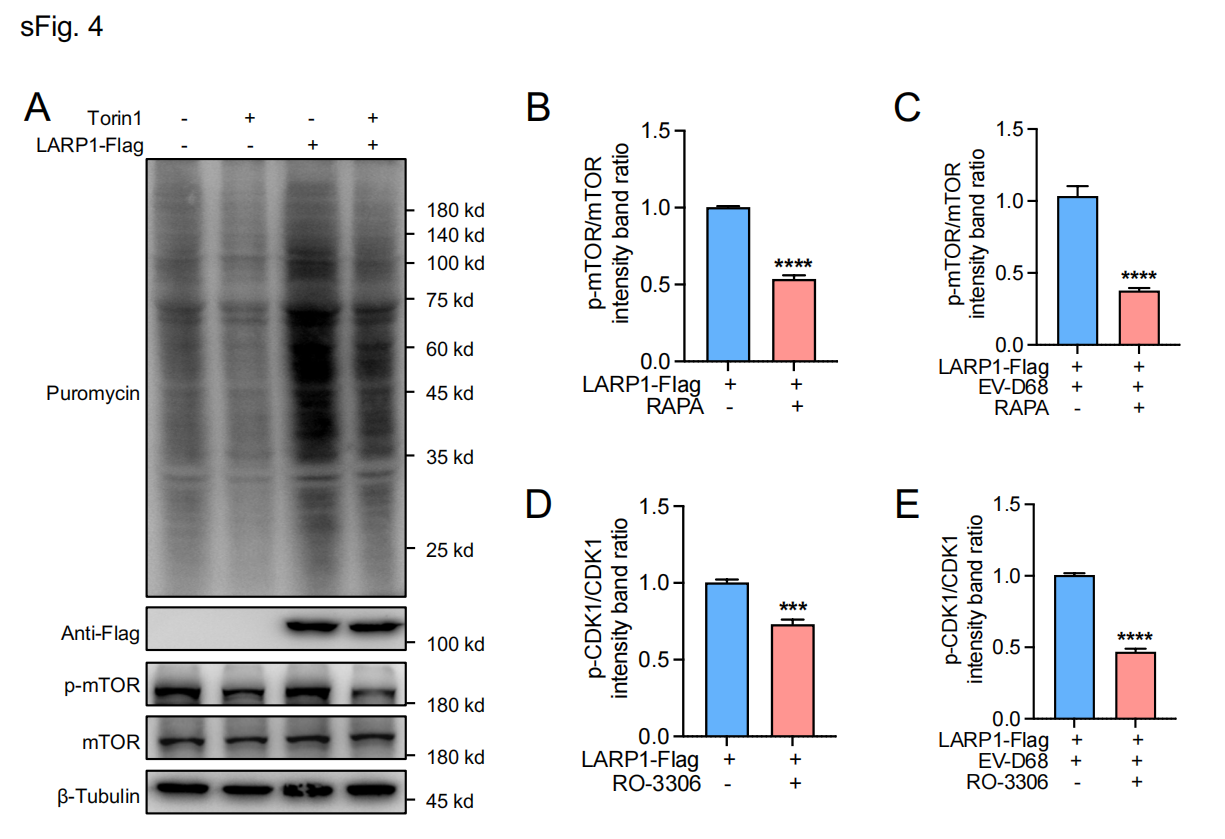

Supplement: S4 Fig — Cells were labelled with 10 µg/mL puromycin for 0.5 h, and then collected for western blot. (B) HEK293T cells were transfected with LARP1-Flag plasmid for 24 h, followed by treatment with or without RAPA (10 µ M) for 12 h. The phosphorylation level of mTOR was analyzed by western blot, and the p-mTOR/mTOR band intensity ratio was quantified. (C) HEK293T cells were co-transfected with LARP1-Flag plasmid and infected with EV-D68 (MOI = 0.1) for 12 h, followed by treatment with or without RAPA (10 µ M) for 12 h. Western blot analysis was conducted to examine mTOR phosphorylation levels, and the p-mTOR/mTOR band intensity ratio was quantified. (D) HEK293T cells were transfected with LARP1-Flag plasmid for 24 h, followed by treatment with or without RO-3306 (10 µ M) for 12 h. Western blot analysis was performed to assess CDK1 phosphorylation levels, and the p-CDK1/CDK1 band intensity ratio was quantified. (E) HEK293T cells were co-transfected with LARP1-Flag plasmid and infected with EV-D68 (MOI = 0.1) for 12 h, followed by treatment with or without RO-3306 (10 µ M) for 12 h. The phosphorylation level of CDK1 was analyzed by western blot, and the p-CDK1/CDK1 band intensity ratio was quantified. (TIF) [file ppat.1013098.s004.tif]
